# Local-Ternary-Pattern-Based Associated Histogram Equalization Technique for Cervical Cancer Detection

**DOI:** 10.3390/diagnostics13030548

**Published:** 2023-02-02

**Authors:** Saravanan Srinivasan, Aravind Britto Karuppanan Raju, Sandeep Kumar Mathivanan, Prabhu Jayagopal, Jyothi Chinna Babu, Aditya Kumar Sahu

**Affiliations:** 1 Department of Computer Science and Engineering, Vel Tech Rangarajan Dr. Sagunthala R&D Institute of Science and Technology, Chennai 600062, India; 2 Department of Electronics and Communication Engineering, PSNA College of Engineering and Technology, Dindigul 624622, India; 3 School of Information Technology and Engineering, Vellore Institute of Technology, Vellore 632014, India; 4 Department of Electronics and Communications Engineering, Annamacharya Institute of Technology and Sciences, Rajampet 516126, India; 5 Amrita School of Computing, Amrita Vishwa Vidyapeetham, Amaravati Campus, Amaravati 522503, India

**Keywords:** cervigram, associated histogram equalization technique, finite ridgelet transform, gray-level run-length matrices, morphological operation, enhanced local ternary pattern

## Abstract

Every year, cervical cancer is a leading cause of mortality in women all over the world. This cancer can be cured if it is detected early and patients are treated promptly. This study proposes a new strategy for the detection of cervical cancer using cervigram pictures. The associated histogram equalization (AHE) technique is used to improve the edges of the cervical image, and then the finite ridgelet transform is used to generate a multi-resolution picture. Then, from this converted multi-resolution cervical picture, features such as ridgelets, gray-level run-length matrices, moment invariant, and enhanced local ternary pattern are retrieved. A feed-forward backward propagation neural network is used to train and test these extracted features in order to classify the cervical images as normal or abnormal. To detect and segment cancer regions, morphological procedures are applied to the abnormal cervical images. The cervical cancer detection system’s performance metrics include 98.11% sensitivity, 98.97% specificity, 99.19% accuracy, a PPV of 98.88%, an NPV of 91.91%, an LPR of 141.02%, an LNR of 0.0836, 98.13% precision, 97.15% FPs, and 90.89% FNs. The simulation outcomes show that the proposed method is better at detecting and segmenting cervical cancer than the traditional methods.

## 1. Introduction

**Figure 1:**
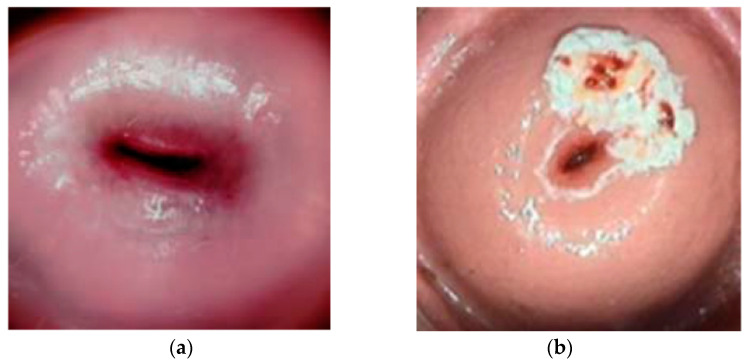
(**a**) Image of normal cervix; (**b**) image of cervix showing abnormal tissue growth.

Cervical cancer is the second most common cancer in women worldwide, with a mortality rate of 60%. Cervical cancer begins with no overt signs and has a long latent period, making early detection through regular checkups vitally important. In this study, we compare the performance of two different models, machine learning and deep learning, for the purpose of identifying signs of cervical cancer using cervicography images. [1]. In a study by Chang et al., innovative data mining approaches for recurrent cervical cancer survival analyses were used. The medical records and pathology were obtained from the Chung Shan Medical University Hospital Tumor Registry. Twelve variables were studied after a literature review, expert consultation, and data collection from patients, including age, cell type, tumor grade, tumor size, pT, pStage, surgical margin involvement, LNM, number of fractions of other RT, RT target summary, the sequence of locoregional therapy and systemic therapy, and LVSI [2]. Adjuvant therapy for patients with intermediate-risk cervical carcinoma (CC) remains unclear. A study by Chu aimed to examine the prognoses of patients with early-stage CC who had pathological characteristics of intermediate risk and to provide a reference for adjuvant therapy selection [3]. Magnetic resonance imaging is used to evaluate the different parts of the brain and study the brain tissues. In the medical image processing arena, in a previous work we offered a method called convolutional neural network database learning with neighboring network limitation (CDBLNL) for brain tumor picture classification. The suggested system architecture was built with multilayer-based metadata learning and has a CNN layer to offer reliable information [4]. Basic research has also been conducted on cervical cancer detection using an optical sensor and a prediction system. Because each substance has a refractive index, monitoring this index and detecting variations in its value provides information about a tissue’s status. Datasets from the optical measurements were used to train and validate the analysis program. Another work provided data pre-processing and machine learning findings using four algorithms (random forest, extreme gradient boosting, naive Bayes, and convolutional neural network), as well as an evaluation of their performance in classifying tissue as healthy or sick [5]. Early-stage cervical cancer is treated with radical hysterectomy. However, this surgery is associated with considerable morbidity as a result of parametrium ablation. PMI is identified in a small percentage of patients, but there is no effective system in place to forecast it. The proposed a novel machine learning (ML)-based predictive model (named iPMI) based on a random forest model for the practical detection of PMI in women [6]. Because cancer cells can grow everywhere, they can develop anywhere, penetrate the walls of arteries and lymph vessels, and aggressively infiltrate other parts of the body. Figure 1 shows a typical uterine cervix image of normal and abnormal cases. There are several varieties of cancer, including skin cancer, breast cancer, lung cancer, and others. The cervix is an important organ in women that generates mucus to help in sexual intercourse [7].

Cervical cancer has surpassed breast cancer as the third most frequent type of cancer worldwide. The majority of cervical cancer cases are connected to the risk of infection with human papillomavirus. Preventive care, the most expensive method of fighting cancer, can prevent approximately 37% of cancer cases. The pap smear test is a routine screening tool for the early detection of cervical cancer. However, due to individual flaws, this manual test process produces a high number of false-positive results. ML (machine learning) methods for classifying cervical papillomatous cells have been studied in depth by a number of academics in order to improve manual testing [8]. It is heartening to note that the world has reached a strategic agreement on cervical cancer eradication, and has established and launched a worldwide plan to expedite cervical cancer elimination. Although there is still a long way to go towards the worldwide elimination of cervical cancer, it is expected that via the contiguous promotion and widespread implementation of existing efficient preventive and control strategies, cervical cancer could become the first cancer abolished by humans [9].

## 2. Related Works

As mentioned previously, cervical cancer is one of the leading causes of cancer deaths in women. If this cancer is detected and treated at an early stage, its complications can be minimized. In this study, we present a cervical cancer cell detection and classification system based on a convolutional neural network (CNN). To extract deep-learning features, the cell pictures are loaded into a CNN model. The input photos are then classified by an extreme learning machine (ELM)-based classifier. Transfer learning and fine tuning are utilized to implement the CNN model. Alternatives to ELM include multi-layer perceptron (MLP)- and autoencoder (AE)-based classifiers. The Herlev database is used for experiments in [10]. Women in developing countries often cannot participate in adequate screening programmes due to the high expense of frequent examinations, a lack of knowledge, and a lack of access to medical facilities. As a result, the risk for individual patients becomes quite significant. There are several risk factors for malignant cervical cancer development. Carcinoma has displaced breast cancer as the third most frequent type of cancer worldwide [11]. Tests such as pap smears require laboratories to identify malignancy from a network of cervical cells. The IVA test uses acetic acid fluid, whereas colposcopy involves assessing the status of the vulva and the vagina and recording it in colposcopy photo data. Photos from colposcopy can be automatically detected using computer-aided diagnosis (CAD) by applying image processing and classifying them using artificial intelligence approaches. The early detection of cervical cancer based on cancer stage using texture information in colposcopy images is investigated in this study, which examines pixel neighbor information using the gray-level co-occurrence matrix (GLCM) method and classifies it using the kernel extreme learning machine (KELM) method, which is a development of the ELM method, adding a kernel to the system [12]. Microscopic examination of skin lesions is the primary method for detecting skin cancer. Significant work has gone into developing computer-aided technologies for analyzing skin lesions. To better analyze and classify skin lesions for diagnosis, one group developed a method for an algorithm design using support vector machine (SVM) learning classification based on particle swarm optimization (PSO) principles [13]. However, because of individual differences, this manual test approach produces a high number of false-positive results. A rising number of people and businesses are turning to machine learning to analyze vast volumes of data and deliver meaningful insights using machine and deep learning [14]. The goal of another study was to develop a machine-learning-based model that incorporates these risk factors into cervical cancer prognosis and prediction. Data on cytokine gene variants, normal healthy controls, and cervical cancer cases were all included in the analysis. Machine learning methods were used to examine a wide variety of potential risk factors, such as demographic information and cytokine gene variants. The proposed method was tested with several statistical measures. Machine learning techniques were applied to the data, evaluated using 5-fold cross-validation, and then tested on the unseen records of a collected dataset to ensure accuracy in evaluation and analysis [15]. Traditional, manual, and human-powered methods are still used by most of the healthcare industry. These methods are hard to use, take a long time, and often result in mistakes. The current paradigm, the chances of new scientific discoveries, the current state of technology, the chances of supervised machine learning (SML) in different areas of healthcare, and ethical concerns. Disease diagnosis, personalized medicine, clinical trials, non-invasive image analysis, drug discovery, patient care services, remote patient monitoring, hospital data, and nanotechnology are evaluated in various learning-based automation tools in healthcare, as is the need for explainable artificial intelligence (AI) in healthcare [16]. The identification of any illness improves a patient’s chances of effective therapy compared to disease detection at a later stage of development. Even if model designers do not know how to treat patients, early diagnosis provides the opportunity for treatment that could be beneficial and make life more comfortable for patients [17]. In the preclinical imaging of patient-derived tumor xenografts (PDXs), magnetic resonance imaging (MRI) is usually used to find and measure how well a treatment is working. The main goal was to develop a way to automatically find and divide tumors in PDXs so that they can be studied further. Automated segmentation reduces user bias, which is beneficial. From volumetric MR images, tumor volume was found and divided using a hybrid method that combined fast k-means, morphology, and level setting. The local density peak estimation method was used to choose the initial centers of k-means. A new variational model was used to take advantage of the information about the region by minimizing the energy function in level set. The mask-specific initialization method was used to create a true boundary for the level set. The performance of tumor segmentation was compared with manually segmented images and with algorithms that had already been used [18]. Timely detection of carcinoma improves recovery rates and lowers mortality rates [19]. Skin cancer is one of the most common types of cancer worldwide. Dermatoscopic images can be used to find it. In a paper by Srivastava et al., the authors develop a method to classify dermatoscopic images using a texture-based feature extraction algorithm. After obtaining a local ternary pattern based on the median, local quantized ternary patterns are made. A modified convolutional neural network is then used to classify the set of extracted features. Images used to find multiple types of skin cancer came from HAM10000 and ISICUDA11 datasets, which are both available to the public [20]. Cancerous cells can be found in their early stages when screening tests are performed on a regular basis, lowering the death rate of individuals each year [21]. Carcinoma is among the most lethal illnesses in women worldwide. It is caused by a long-term infection in the vaginal skin and mucous membrane cells. The most concerning aspect of this cancer is that it has no symptoms when it first appears [22]. The proposed model has been used for predicting the proper stage of infection in breast cancer. In recent decades, the computer-aided classification of smear pictures has been regarded as difficult task [23]. Computerized image analysis technologies are particularly valuable because they give major benefits to doctors by providing reliable and quick diagnosis of data [24]. Table 1 describes the various techniques utilized in state-of-the-art methods and their outcomes.

## 3. Proposed System

### 3.1. Materials

The cervical images in this paper are from the ARC Cervical Cancer Image Bank [25]. It is a global open-access image database and comprises a huge number of cervical pictures, each with an equivalent ground-truth image annotated by an expert clinician. The images from this collection are dynamically sorted into several sensitivity classifications in this study.

### 3.2. Methods

**Figure 2:**
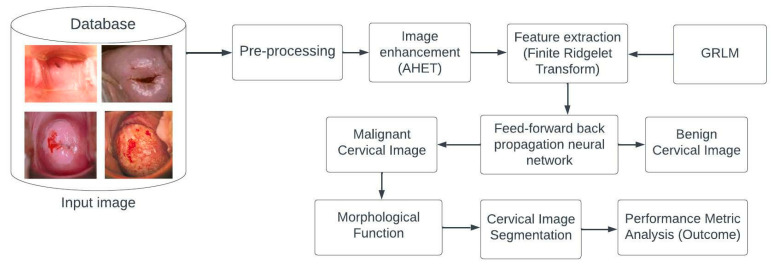
Proposed system block diagram.

A computer-aided automatic detection system is the proposed method for detecting cervical cancer. Figure 2 depicts the overall process for the detection mechanism. The original cervical image is first pre-processed using the associated histogram equalization technique for image enhancement, after which the enhanced image is transformed using the finite ridgelet transform. Then, features like ridgelets, gray-level run-length matrices, moment invariant, and enhanced local ternary pattern are extracted from the pre-processed image. By comparing the cervical image with the trained features, the neural network classifier is trained to classify the tumor as benign or malignant. Performance metric parameters such as sensitivity, specificity, and accuracy are used to analyze the cervical image classification.

#### 3.2.1. Associated Histogram Equalization Technique

The pre-processed image is used to extract features including ridgelet, GRLM, and phase-independent features. When compared to the learned characteristics, these features are utilized to build the neural network classification model to categorize the cervical pictures as benign or cancerous. In this article, pre-processing is performed to improve the interior regions of the cervical image in order to achieve significant irregular section segmentation. The cervix pictures are in RGB format, and are transformed to grayscale for more processing.

Improvement is also necessary for poor-quality cervical images, to enhance the image edges. The AHE improvement approach is employed in this work to enhance contrast in cervical pictures. This approach is a variation on limited histogram equalization (LHE), differing in that it also records edge orientation. AHE is used to calculate for each pixel of a cervical picture utilizing a local window centered on the given pixel, as

(1) $$s\left(a\right)=round_{i}\left[\frac{Cumulative\;distribution\;function\;\left(a\right)-Cumulative\;distribution\;function_{min}}{width*height-Cumulative\;distribution\;function_{min}}(L_{i}-1\right)].$$

Here, (*a*) in Equation (1) is the value of pixel intensity, cumulative distribution function (*a*) is the function of histogram equalization of the intensity of the pixel value, the dimensions (width × height) of the window are generally 3 × 3 pixels for starting and ending points of the image, the min value is the cumulative distribution function’s lesser intensity value of the window, and $L_{i}$ is the gray-level outcome. This value is floating, resulting in pixel losses during reconstruction. To circumvent this, the improved pixel value is rounded to the nearest integer. At the beginning of the image, the window is shifted from right to left, ending with the final pixel in the cervical image. Improved pixels are created with each window movement. The anchor is the center of the k × k window. The anchor point for AHE is the pixel to be processed. The formal definition of the AHE operators is given below:

(2) $$Top\;Left\;\left(TL\right)=M_{n}^{\left(\frac{\left(n-1\right)}{2},\;\frac{-\left(n-1\right)}{2}\right)}$$

(3) $$Top\;Right\;\left(TR\right)=M_{n}^{\left(\frac{-\left(n-1\right)}{2},\;\frac{-\left(n-1\right)}{2}\right)}$$

(4) $$Top\;Center\;\left(TC\right)=M_{n}^{\left(0,\;\frac{-\left(n-1\right)}{2}\right)}$$

(5) $$Center\;Right\;\left(CR\right)=M_{n}^{\left(\frac{-\left(n-1\right)}{2},\;0\right)}$$

(6) $$Center\;Left\;\left(CL\right)=M_{n}^{\left(\frac{\left(n-1\right)}{2},\;0\right)}$$

(7) $$Lower\;Left\;\left(LL\right)=M_{n}^{\left(\frac{\left(n-1\right)}{2},\;\frac{\;\left(n-1\right)}{2}\right)}$$

(8) $$Lower\;Right\;\left(LR\right)=M_{n}^{\left(\frac{-\left(n-1\right)}{2},\;\frac{-\left(n-1\right)}{2}\right)}$$

(9) $$Lower\;Center\;\left(LC\right)=M_{n}^{\left(0\;,\;\frac{-\left(n-1\right)}{2}\right)}$$

**Figure 3:**
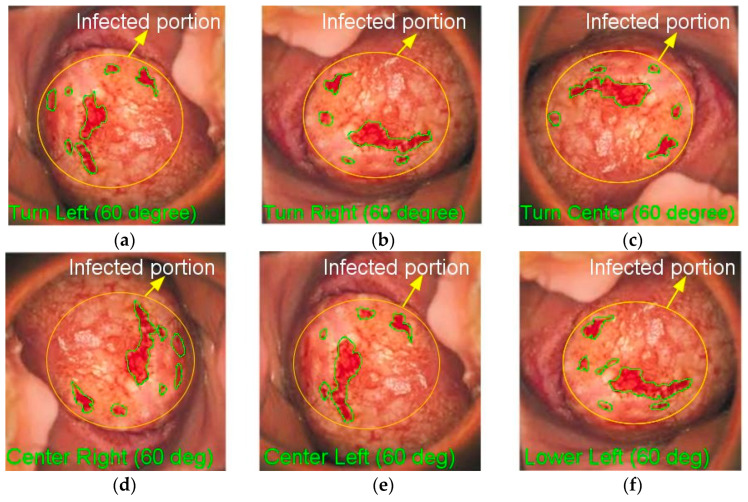
Cervical cancer 60° orientation images: (**a**) TL; (**b**) TR; (**c**) TC; (**d**) CR; (**e**) CL; (**f**) LL.

If *n* is an odd number, during the AHE procedure, eight high-resolution pictures are created. In each contrast-improved image, the higher-intensity pixels are utilized to pick the best pixel intensity to generate the improved image. This approach produces improved cervical pictures in each direction, as seen in Figure 3.

Figure 3 clearly shows that the pixels in the enhanced cervical pictures have greater pixel values than the original cervical images. In the enlarged cervical picture, the aberrant patterns are plainly obvious. The cervical image decomposition can be conducted using the MATLAB software.

#### 3.2.2. Finite Ridgelet Transform

Several image processing tasks make use of sparse image data representations, in which the majority of information is compressed into a limited amount of data. These structures are often obtained via differentiable and nonredundant transformations. The wavelet transform and the discrete cosine transform are now the most common solutions for this purpose. The construction of discrete variants of the ridgelet transform which relate to computational solutions is a difficult challenge for practical uses. Because of the ridgelet’s radial character, simple implementations based on the discretization of continuous equations necessitate interpolation, resulting in transformations that are either redundant or incompletely restored. The function *f(a)* and its finite ridgelet transform in *S^2^* are expressed as:

(10) $$FRT_{f}\left(x,y,\theta \right)=\int_{S^{2}}^{n}\psi_{x,y,\theta }\left(a\right)f\left(a\right)da.$$

Here, the ridgelets $\psi_{x,y,\theta }\left(a\right)$ in two dimensions are expressed from a one-dimensional wavelet-based function ψ(a),

(11) $$\psi_{x,y,\theta }\left(a\right)=x^{-\frac{1}{2}}\psi \left(\left(\left(a_{1}\;cos\theta +a_{2}\;sin\theta -y\right)\right)/x\right).$$

**Figure 4:**
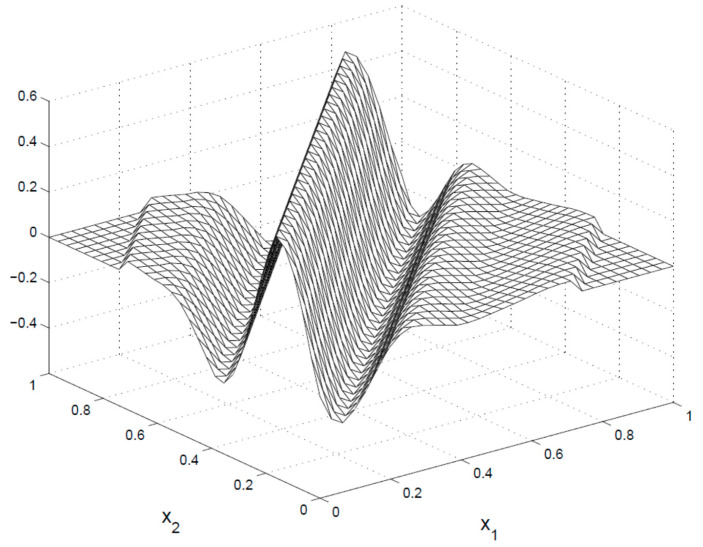
Finite ridgelet function operation.

The finite ridgelet function is shown in Figure 4, and it has an orientation angle *θ* with constant given as $a_{1}\;cos\theta +a_{2}\;sin\theta =const.$

The separable finite transform in *S^2^* of f(a) is written as,

(12) $$FRT_{f}\left(x_{1},x_{2},y_{1},y_{2}\right)=\int_{S^{2}}^{n}\psi_{x_{1},x_{2},y_{1},y_{2}}\left(a\right)f\left(a\right)da.$$

Here the ridgelets in two-dimensional tensor components are

(13) $$\psi_{x_{1},x_{2},y_{1},y_{2}}\left(a\right)=\psi_{x_{1},y_{1}}\left(a_{1}\right)\psi_{x_{2},y_{2}}\left(a_{2}\right).$$

Wavelets excel at expressing things with isolated point singularities, whereas ridgelets excel at representing objects with singularities along lines. Ridgelets may be thought of as a technique for concatenating one-dimensional wavelets along lines. As a result, the justification for employing ridgelets in image processing tasks is compelling, because singularities in pictures are frequently connected along edges or contours.

(14) $$R_{o}\left(\theta ,n\right)=\int_{S^{2}}^{t}f\left(a\right)\vartheta \left(a_{1}cos\theta +a_{2}sin\theta -n\right)da.$$

(15) $$FRT_{f}\left(x,y,\theta \right)=\int_{S^{2}}^{\propto }\psi_{x,y}\left(n\right)R_{o}\left(\theta ,n\right)dn.$$

This typical ridgelet transform is a one-dimensional wavelet transform applied to Radon transformation slices.

#### 3.2.3. Enhanced Local Ternary Pattern (ELTP)

**Figure 5:**
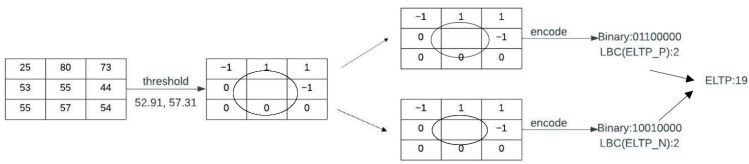
Process of enhanced local ternary pattern.

Local binary pattern [26] handles rotation-invariant texture classification by completely rejecting any microstructure that is not absolutely rotation-invariant under large lighting variations. To solve the shortcomings of LBPs, the suggested enhanced local ternary pattern is adopted herein. The gray level in a zone (tolerance) of width $\pm w^{e}$ around $a_{c}^{e}$ is quantized to 0, and is then further quantized to +1 and −1. The local binary pattern is replaced by

(16) $$T^{e}\left(a_{p},a_{c}^{e},w^{e}\right)=\{\begin{array}{c} 1,a_{p}-a_{c}^{e}\geq w^{e}\\ 0,\left|a_{p}-a_{c}^{e}\right|<w^{e}\\ -1,a_{p}-a_{c}^{e}\leq -w^{e} \end{array}$$

where $a_{c}^{e}$ = mean, $w^{e}$= mad, $A=\left\{a_{i}\left|j=0,1,2,3\ldots n\right|\right\}$. The qualities of the pattern of pixels in the image are represented by features. In this research, features such as GRLM, moment-invariant features, wavelet features, and enhanced local ternary pattern features are recovered from Gabor converted cervical images to distinguish between normal and pathological cervical images. To keep things simple, the experiments employ a coding scheme that divides each ternary pattern into its positive and negative halves, as shown in Figure 5, before merging the two different channels of LBC definitions to form the final improved LTP descriptor and computing the histogram and correlation matrix. Naturally, improved LTP is rotation-invariant.

#### 3.2.4. Gray-Level Run-Length Matrices

One of the most difficult tasks in image processing is texture categorization under different lighting conditions. This work provides a novel texture classification method based on the application of robust illumination normalization techniques to a gray-level run-length matrix (GLRLM) for the extraction of texture information. The GLRLM was chosen as a texture descriptor because it collects information from an image’s gray-level transitions. A gray-level run is a series of successive, collinear image points with the same gray-level values. Variations in light and camera attitude frequently cause a significant shift in the pictures of textured materials. For example, keeping all parameters fixed but altering size and rotation might result in an entirely different texture. As a result, the gray-level values change. The gray-level run is a cluster of image points that are linearly nearby and have comparable gray-level values. A run-length matrix *M* is defined as follows: each element *M (x, y)* represents the number of runs with pixels of a given gray-level intensity, and *y* is the length of a cycle in a specified orientation. Matrix *M* has dimensions *p × q*, where *p* is the highest gray level in the image and *q* is the greatest viable run length in the corresponding image.

(17) $$\begin{array}{ccc} SHE=\frac{1}{len}\underset{a,b}{\sum }\frac{M\left(a,b\right)}{b^{2}} & LOE=\frac{1}{len}\underset{a,b}{\sum }M\left(a,b\right) & GLNU=\frac{1}{len}\underset{a}{\sum }{\left(\underset{b}{\sum }M\left(a,b\right)\right)}^{2}\\ RLNU=\frac{1}{len}\underset{a}{\sum }{\left(\underset{a}{\sum }M\left(a,b\right)\right)}^{2} & RUP=\underset{a,b}{\sum }\frac{1}{M\left(a,b\right)b} & LGLRE=\frac{1}{len}\underset{a,b}{\sum }\frac{M\left(a,b\right)}{b^{2}}\\ HGLRE=\frac{1}{len}\underset{a,b}{\sum }a^{2}M\left(a,b\right) & 1 & 1 \end{array}$$

The orientation is specified using a displacement vector *x (a, b)*, where *a* and *b* are the displacements for the *x*- and *y*-axes, respectively. Four orientations (0°, 45°, 90°, and 135°) are utilized to create texture runs in this method, and four run-length matrices are formed as a result. GLRLM is used to generate seven features: short run emphasis (SHE), long run emphasis (LOE), gray-level non-uniformity (GLNU), run-length non-uniformity (RLNU), run percentage (RUP), low-gray-level run emphasis (LGLRE), and high-gray-level run emphasis (HGLRE).

#### 3.2.5. Moment Invariant Features (MIF)

The moment invariants approach is used to extract the input features. Moment invariants are classified into several categories, including Legendre, geometric, Zernike, and nonlinear moment invariants. Legendre moment invariants were chosen because their performance is superior to others. They are employed in the cervical images for pattern recognition. Normalization is accomplished by the application of complex and geometric moment invariants.

(18) $$\partial_{a,b}=\sum_{p}\sum_{q}{\left(p-\bar{p}\right)}^{a}\cdot {\left(q-\bar{q}\right)}^{b}f\left(p,q\right).$$

**Figure 6:**
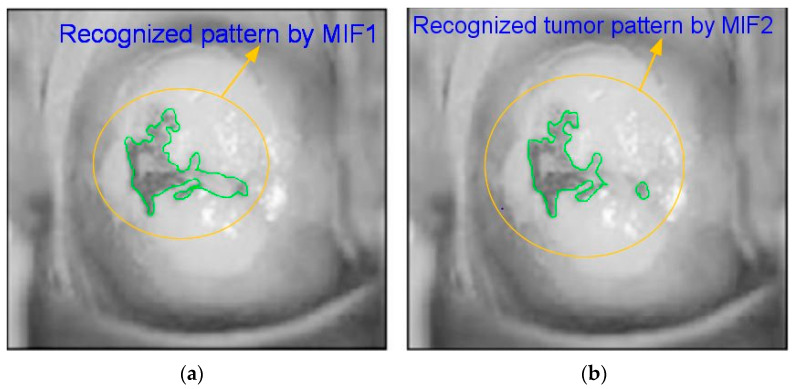
(**a**) MIF image 1; (**b**) MIF image 2.

Figure 6a,b illustrate the different extracted moment invariant feature images and Equation (18) is the MIF equation.

#### 3.2.6. Morphological Function

Cancer areas are segregated utilizing morphological procedures on aberrant cervical images. The erosion and dilation of the set with a structuring element are morphological operators. Releasing is the erosion followed by dilation of a picture; it fractures narrow isthmuses and removes tiny items and sharp peaks from the image. Closing, on the other hand, is picture dilation followed by erosion; it fuses thin cracks and fills microscopic holes and gaps in the image. By eliminating and adding small shapes in the focused photos, this approach may properly increase the regions of interest (ROIs) in images. Dilation is used on the identified abnormal cervical pictures to gradually increase the limits of foreground pixel areas.

(19) $$D_{i}⊕r=\left\{a\left|[({\hat{r})}_{a}\cap D_{i}\right|\right\}.$$

(20) $$D_{i}⊙r=\left\{a\left|[{\left(r\right)}_{a}\subseteq D_{i}\right|\right\}.\;$$

**Figure 7:**
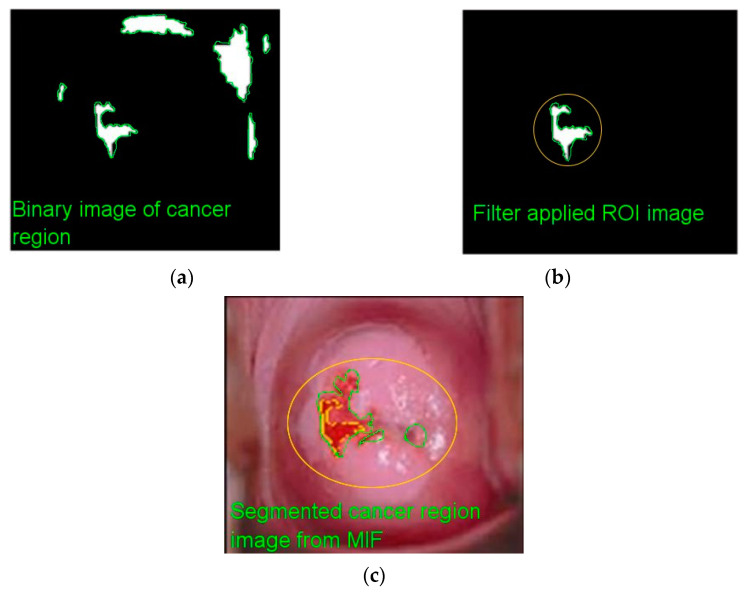
Different images: (**a**) binary; (**b**) ROI; (**c**) cancer segmented.

Erosion function is used on the categorized abnormal cervical picture to erode the borders of foreground pixel areas. Figure 7 illustrates the different image types (e.g., binary image, ROI image, cancer segmented image).

## 4. Results and Discussion

**Figure 8:**
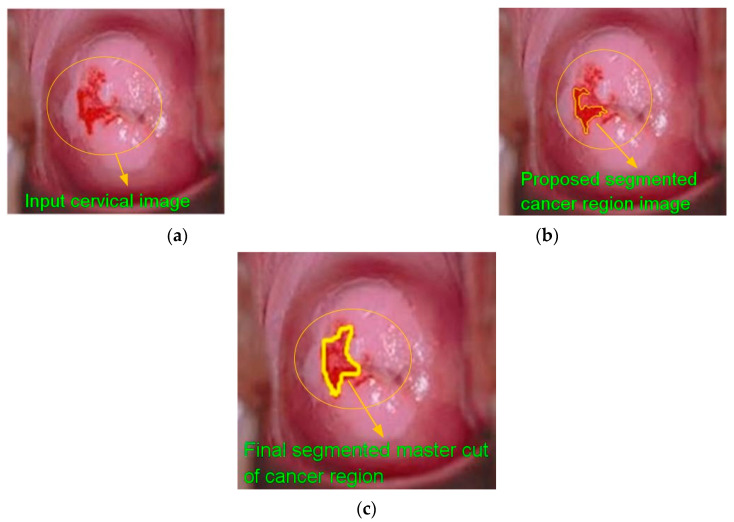
Severe (**a**) cervical; (**b**) cancer segmentation outcome; (**c**) gold typical image.

The proposed cervical cancer detection system’s performance is assessed using a confusion matrix of size 2 × 2, and the values of true positive, true negative, false positive, and false negative are estimated with regard to ground-truth pictures collected from an experienced radiologist. The average sensitivity, specificity, accuracy, positive predictive value, negative predictive value, positive likelihood ratio, and negative likelihood ratio are determined, and the essential definitions of these performance metrics are explained below. All these performance metric indicators are expressed as percentages. Table 2 summarizes the performance evaluation results. Figure 8 clearly shows the findings of the categorized cervical cancer images with cancer stages (e.g., normal and segmented instances).

**Figure 9:**
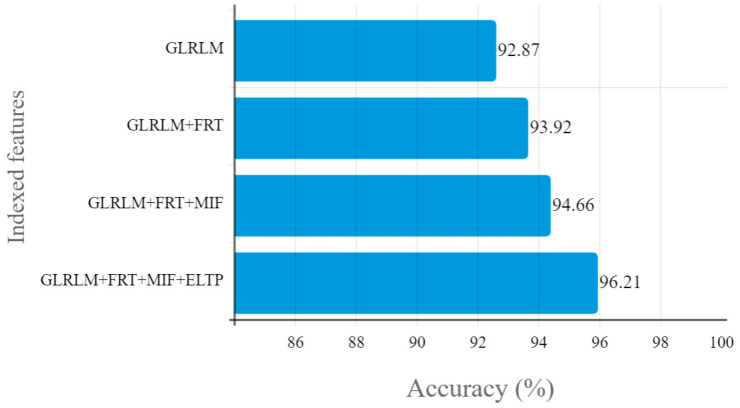
Graphical illustration of the accuracy results of cervical cancer segmentation.

Table 3 illustrates the feature indexed accuracy values of cervical cancer segmentation. The proposed system obtained 92.87% accuracy with GLRLM features, 93.92% accuracy with GLRLM + finite ridgelet transform features, and 94.66% accuracy with GLRLM + finite ridgelet transform + moment invariant features. Finally, the proposed system obtained 96.21% accuracy with GLRLM + finite ridgelet transform + moment invariant features + enhanced local ternary pattern. Figure 9 depicts a graphical illustration of the cervical cancer segmentation accuracy outcomes of different features.

**Figure 10:**
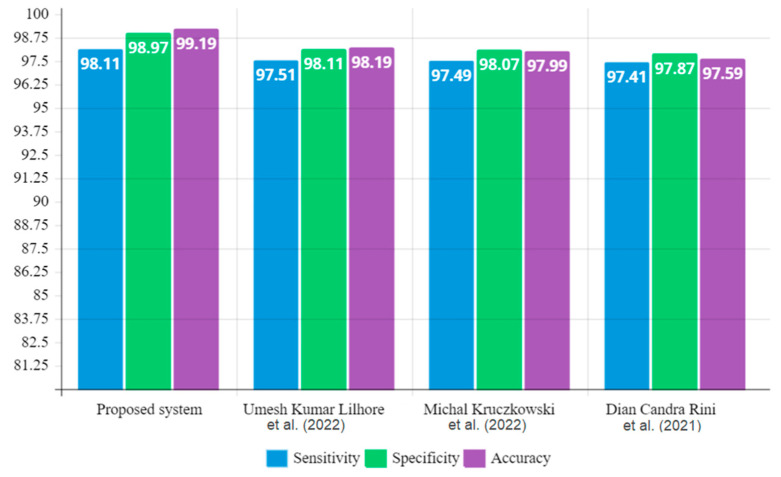
Graphical illustration of performance metric comparison outcomes.

Table 4 summarizes the performance metric comparison of the proposed cervical cancer segmentation technique and other traditional methods; the proposed system obtained superior performance metric outcomes to other methods, yielding 98.11% sensitivity, 98.97% specificity, and 99.19% accuracy. Figure 10 depicts a graphical illustration of performance metric comparison outcomes of the proposed method and existing methods. In this paper, the proposed model is tested using k-fold cross-validation. In this method of validation, the total number of images of the cervical region is divided into *k* equal numbers of sample data. The first set of sample data from a set of *k* samples is used for validation, and the other *k*-1 sample data are used to train the method. In this paper, the results of the proposed method are checked using two-fold cross-validation with *k* = 2. The following equation is used to determine the cross-validation error (*µ*) between *k* samples:

(21) $$\mu =\frac{1}{k}\sum_{k=1}^{K}C\left(k\right).$$

Here, *k* is set to 2 to reduce the error in cross-validation between samples after many trials. The error in cross-validation is between 0 and 1. A low cross-validation error indicates that a method is the best one for testing, while a high cross-validation error indicates that a method is not the best for testing. The most important goal of this study on cancer segmentation is to determine the best performance evaluation parameters for determining the severity of cancer in each area with an automated process.

## 5. Conclusions

For the purpose of detecting cancer from cervical images, an automated detection and classification method is proposed that makes use of a set of biologically and clinically relevant features. A convolutional neural network is used to detect and classify the cancer regions. The associated histogram equalization technique is used to enhance the cervical images. A neural network classifier is used to classify the cervical images into normal and abnormal images. The simulation results demonstrated that the proposed scheme for detecting benign and cancerous regions in cervical images obtained better outcomes than existing methods. The cervical cancer detection system achieved 98.11% sensitivity, 98.97% specificity, 99.19% accuracy, a PPV of 98.88%, an NPV of 91.91%, an LPR of 141.02%, an LNR of 0.0878, 98.13% precision, 97.15% FPs, and 90.89% FNs. This method can be developed in the future to classify segmented cancer areas in cervical imaging as “Early” for treatment to prevent death. In the future, cervical imaging and pap smear images may also be used to determine how this cancer affects other disorders.

## Figures and Tables

**Table 1:** Different state-of-the-art-methods.

Author Name	Technique	Obtained
Ghoneim et al. (2020) [10]	ELM-, multi-layer perceptron (MLP)- and autoencoder (AE)-based classifiers	Using the Herlev database, the proposed system with the ELM-based classifier achieved 98.7% accuracy in the 2-class problem and 97.2% accuracy in the 7-class problem
Dian Candra Rini Novitasari et al. (2020) [12]	Texture information, pixel neighbor information, gray-level co-occurrence matrix and kernel extreme learning machine	Linear kernel resulted in an error of 78.5%, polynomial kernel an error of 87.5% and the best accuracy of 95% was achieved using a gaussian kernel with the best neighborhood angle of 45°
Fei et al. (2020) [13]	Support vector machine, particle swarm optimization	Segmentation was robust because the local extracted features from ROI were acceptable. This technique provides high accuracy to support assisting clinicians in classifying skin lesion images into relevant diagnostic categories
Kaushik et al. (2021) [15]	Five-fold cross-validation, logistic regression	Highest average accuracy of 82.25% and highest average F1-score of 82.58%
Sudipta Roy et al. (2022) [10]	Supervised machine learning	Effectiveness and potential for innovation of disease diagnosis, personalized medicine, clinical trials, non-invasive image analysis, drug discovery
Sudipta Roy et al. (2019) [16]	Patient-derived tumor xenografts, fast k-means, morphology	Segmentation results obtained from six metrics were Jaccard score (>80%), Dice score (>85%), F-score (>85%), G-mean (>90%), volume similarity matrix (>95%)
Varun Srivastava et al. (2022) [20]	Median-based local ternary pattern	The proposed technique, the average recall value, average precision and average accuracy were found to be 75.20%, 95.44%, and 96% respectively
Abbas et al. (2021) [23]	Extremely randomized tree and whale optimization algorithm	BCD-WERT outperformed all with the highest accuracy rate of 99.30% followed by SVM achieving 98.60% accuracy
Simaiya et al. (2021) [24]	Hierarchical k-means clustering with fuzzy c and Super-Rule-Tree	Plus-Rule-Tree to face the issue of misplaced patterns. Proposed method had accuracy of 88.9%, and existing k-means clustering method showed accuracy of 85.4%

**Table 2:** Evaluation outcomes.

Metric Parameters	Estimated Values (%)
Sensitivity	92.17
Specificity	98.92
Accuracy	97.11
Positive Prediction Value	98.88
Negative Prediction Value	91.91
Positive Likelihood Ratio	141.02
Negative Likelihood Ratio	0.0878
Precision Rate	98.13
False Positive	97.15
False Negative	90.89

**Table 3:** Accuracy result of cervical cancer segmentation.

Indexed Features	Accuracy (%)
GLRLM	92.87
GLRLM+FRT	93.92
GLRLM+FRT+MIF	94.66
GLRLM+FRT+MIF+ELTP	96.21

**Table 4:** Performance metric comparison of proposed and existing techniques.

Technique/Method	Parameters		
	Sensitivity (%)	Specificity (%)	Accuracy (%)
Proposed system	98.11	98.97	99.19
Umesh Kumar Lilhore et al. (2021) [24]	97.51	98.11	98.19
Michał Kruczkowski et al. (2022) [5]	97.49	98.07	97.99
Dian Candra Rini Novitasari et al. (2020) [12]	97.41	97.87	97.59

## Data Availability

Not applicable.
